# Transformation of Abdominal Wall Endometriosis to Clear Cell Carcinoma

**DOI:** 10.1155/2015/123740

**Published:** 2015-09-17

**Authors:** Maria Paula Ruiz, Darryl Lewis Wallace, Matthew Thomas Connell

**Affiliations:** Department of Obstetrics and Gynecology, University of Missouri School of Medicine, 2301 Holmes Street, Kansas City, MO 64064, USA

## Abstract

Clear cell carcinoma is the least common of the malignant transformations reported in nonpelvic sites of endometriosis. Two cases with clear cell carcinoma transformation arising from endometriosis in abdominal wall scars are presented. These patients underwent total abdominal hysterectomy with bilateral salpingo-oophorectomy, pelvic washings, and abdominal wall lesion resection. The first case had initial treatment with chemotherapy, while chemotherapy and radiation therapy were given for the second case. A recurrence was noted for the chemotherapy only case for, which she was subsequently given radiation, with further resolution of the lesion.

## 1. Introduction

Endometriosis is a common benign diagnosis in women, yet its extraperitoneal presence, while well described, is rare. In the past, encountering endometrial implants in cesarean section scars was an uncommon event [1]. However, as the rates of cesarean section have increased, these findings have been reported with increased frequency. The risk of transformation of endometriosis to a malignant disease is currently thought to be in the range of 0.3–1.0% [2]. Clear cell carcinoma of the ovary is the least predominant type of epithelial ovarian cancer representing only 6% of all histologic types [3]. An association of endometriosis as a precursor to clear cell and endometrioid carcinoma has been postulated in the current model of ovarian carcinoma [4]. We present two cases of clear cell carcinoma arising from endometriosis within cesarean section scars.

## 2. Case Reports

### 2.1. Report #1

A 41-year-old African-American female, gravida 4, para 4, presented for evaluation of heavy vaginal bleeding and abdominal pain. Her history was significant for a cesarean section 20 years ago. Physical exam was significant for a 2 cm cystic tender lesion under the Pfannenstiel scar. Pelvic ultrasound demonstrated a 9 cm uterus with a submucosal fibroid and a 3.1 × 3.5 × 3.5 cm simple cyst on the left ovary. Endometrial biopsy demonstrated an endometrial polyp. Diagnostic hysteroscopy with ablation was performed for endometrial polyp evaluation and treatment. CA-125 was 22. The Pfannenstiel mass was evaluated with a CT of the abdomen and pelvis, which showed a 4.5 × 5.6 × 8.1 cm heterogeneous soft tissue mass with a fluid component within the rectus abdominis muscle and subcutaneous fat.

**Figure 1:**
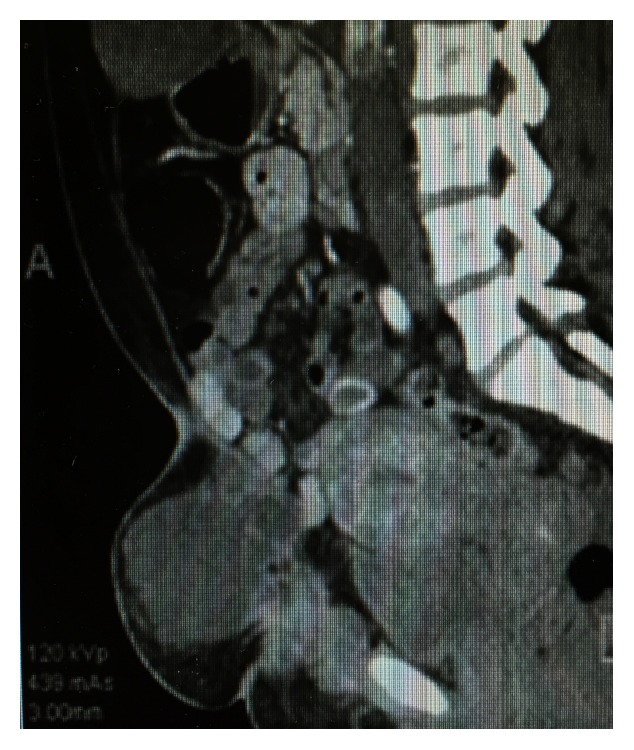
CT abdomen and pelvis demonstrating abdominal mass.

**Figure 2:**
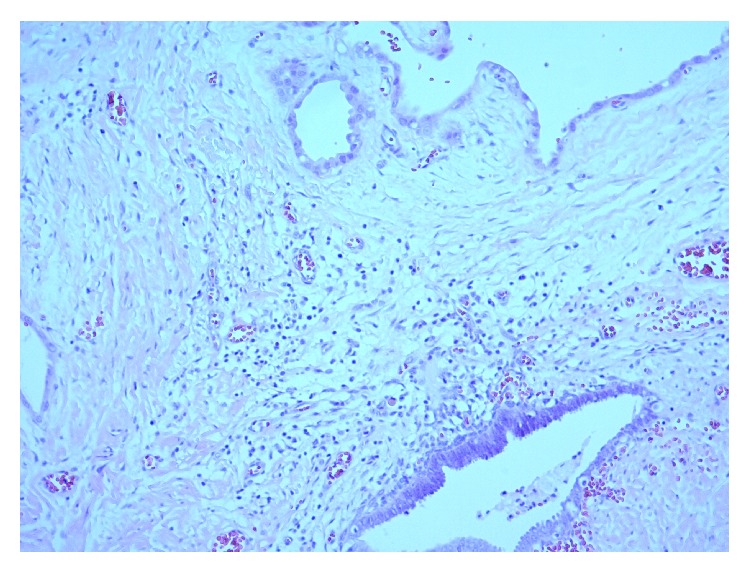
Clear cell carcinoma arising from the setting of endometriosis. Hematoxylin and eosin stain, 100x magnification.

The patient was lost to follow up for approximately 1 year, after which she returned with abdominal pain. Abdominal and pelvic CT (Figure 1), along with MRI, showed enlargement of mass to 14.8 × 7.6 × 10.7 cm. A needle biopsy was performed which revealed cystic spaces lined by atypical glandular cells with clear cytoplasm and atypical nuclei, highly suspicious for clear cell carcinoma arising from a setting of endometriosis (Figure 2).

The patient underwent total abdominal hysterectomy with bilateral salpingooophorectomy, partial omentectomy, and pelvic washings along with complete excision of the mass. Histopathologic workup yielded the diagnosis of clear cell carcinoma arising in association with endometriosis from the Pfannenstiel scar lesion. Uterine pathology was significant for adenomyosis with subserosal leiomyomas. The left fallopian tube had evidence of hydrosalpinx and chronic salpingitis. The remaining pathology was without evidence of malignancy.

The postoperative course was complicated by a partial wound separation on postoperative day #38, which healed by secondary intention. Following surgery, she received adjuvant chemotherapy consisting of carboplatin and paclitaxel for a total of 6 cycles. The patient had regular follow-up visits, and at 6 months a CT was obtained for evaluation of a newly found mass at the same location where the initial lesion was excised. CT revealed a new anterior abdominal wall mass within the rectus muscles. A fine needle aspiration was performed and demonstrated atypical cells present suspicious for malignancy. Targeted radiation of the new mass was performed over 6 sessions. A follow-up CT was performed which showed resolution of the mass.

### 2.2. Report #2

A 57-year-old female, gravida 3, para 3, presented for evaluation of a 9-month history of pain under Pfannenstiel scar. Her history was significant for 3 cesarean sections, 30 years ago, with tubal ligation. Physical exam was significant for a tender, 7 cm mass under the Pfannenstiel scar and bilateral inguinal lymphadenopathy. CT and MRI evaluation confirmed an abdominal wall tumor extending to the bladder without invasion. Needle core biopsy of the mass and a right inguinal lymph node revealed poorly differentiated adenocarcinoma, possibly Müllerian in origin. The patient's initial laboratory evaluation revealed a normal CA 19-9. CEA and CA-125 were elevated at 96.5 and 81, respectively. The patient underwent wide local excision of the abdominal wall mass, total abdominal hysterectomy with bilateral salpingooophorectomy, and bilateral inguinal/internal iliac lymphadenectomy with the removal of nodules from the serosa of the terminal ileum and cecum. Frozen section revealed adenocarcinoma of abdominal wall mass without evidence of malignancy in fallopian tubes, ovaries, or uterus. Plastic surgery was required for the closure of the 35 cm abdominal wall defect with biologic mesh and abdominal wall flap.

Final pathology showed the fallopian tubes, ovaries, and uterus without abnormality. The abdominal wall lesion was 19.4 cm in the greatest dimension. Pathology showed poorly differentiated adenocarcinoma with clear cell features and positive margin at pubic symphysis. Findings were consistent with clear cell carcinoma of Müllerian origin, with endometriosis lesions noted in close proximity of the clear cell adenocarcinoma. One inguinal lymph node (6.2 cm) and one internal iliac lymph node (6.1 cm) showed metastasis of clear cell carcinoma.

Her postoperative course was complicated by a small area of wound infection one month after surgery, which was treated with antibiotics. Following recovery, the patient was treated with carboplatin and taxol for 3 cycles due to positive nodes found, followed by radiation therapy to the positive pubic symphysis margin followed by 3 more cycles of chemotherapy. She was followed up during treatment with CA-125 levels, which progressively dropped to below 7. Follow-up evaluation with CT revealed no evidence of recurrence.

## 3. Discussion

Endometriosis has been proposed as a precursor of clear cell carcinoma of the ovary [4]. The time frame to the diagnosis of clear cell carcinoma arising from endometriotic implants in anterior abdominal wall scars ranges from 9 to 35 years after a cesarean section. 19 cases have been reported to date (15 cases noted in reference article [5], 1 case reported by Bats et al. 2008 [6], 1 case by Alberto et al. [7], and 2 cases presented here). Patient ages have ranged from 38 to 60 years at diagnosis [5].

Of the three subtypes of extragonadal malignant endometrial transformations, endometrioid subtypes represent 69.1%, sarcoma subtypes represent 25%, and clear cell carcinoma subtypes represent 4.5% [6]. When evaluating malignant abdominal wall endometriosis, clear cell carcinoma is the most commonly found [6].

Unfortunately, a specific marker for diagnostic purposes has not been identified. Due to rarity, there is no standardized management. Index of suspicion leading to fine needle biopsy is usually diagnostic. In malignant transformations, CA-125 has been reported as normal to mildly elevated [8] with high sensitivity and specificity at 87 and 92%, respectively [9].

From the few cases reported in the literature, it has been noted that the rectus muscle may be intimately involved within the lesion [7] and the uterus may have evidence of adenomyosis, while there may or may not be intraperitoneal endometriosis [6].

Treatment of abdominal wall adenocarcinoma arising from endometriosis is not standardized; therefore, adoption of ovarian cancer management has been routinely performed in these patients. Although there is no consensus or evidence based guidelines for this management, complete surgical resection of the lesion should be performed, with strong consideration for surgical staging similar to ovarian cancer. Of the cases presented here, complete resection of the masses was achieved. To achieve complete resection, it may require radical cytoreductive surgery, which may require reconstruction of the abdominal wall. Groin lymph nodes are the main lymphatic spread from lower abdominal wall disease and may require excision and/or sampling. Cases with bulky disease may warrant neoadjuvant chemotherapy prior to surgery. In the cases described, decision for chemotherapy administration was prophylactic for Case 1 and treatment for lymphatic invasion noted in Case 2. Postoperative radiation therapy to prevent/treat local recurrence may be considered. Recurrences are uncommon but have been reported, including progression to death. The lack of standardized management and treatment of these rare malignant transformations poses the greatest challenge to patient care. The two cases presented will add to the current body of literature. Patients with a remote history of endometriosis may have severe sequelae from their disease. Further investigation is required to understand which patients may be at risk for this transformation and how to best treat them.
